# A case of computed tomography diagnosis of truncus arteriosus type IV

**DOI:** 10.1093/omcr/omaa144

**Published:** 2021-02-15

**Authors:** Abid M Sadiq, Adnan M Sadiq

**Affiliations:** 1 Department of Internal Medicine, Kilimanjaro Christian Medical Centre, Moshi, Tanzania; 2 Department of Radiology, Kilimanjaro Christian Medical Centre, Moshi, Tanzania; 3 Department of Internal Medicine, Kilimanjaro Christian Medical University College, Moshi, Tanzania; 4 Department of Radiology, Kilimanjaro Christian Medical University College, Moshi, Tanzania

## Abstract

Persistent truncus arteriosus is a rare congenital heart disease with four variants, and the last being the rarest. The prognosis without surgical intervention is poor. In such cases, an echocardiography is not sufficient hence computed tomography (CT) imaging is required. We report a 26-year-old female with difficulty in breathing since childhood with cyanosis. Her echocardiography showed a ventricular septal defect (VSD) and the CT showed a single arterial trunk overriding the interventricular septum with a VSD, and the descending aorta giving rise to the pulmonary arteries suggestive of pseudo truncus, known as truncus arteriosus type IV.

## INTRODUCTION

Persistent truncus arteriosus (PTA) is a rare cyanotic congenital heart disease as it accounts for around 1% of all congenital heart diseases (CHD). It is characterized by the truncus arteriosus failing to divide into the aorta and pulmonary arteries during embryonic development, leading to a single vessel with varying characteristics [1].

Prognosis is generally poor as the main management is surgical during infancy but there have been records of survival into adulthood without intervention. Echocardiography may not always be useful in evaluation such CHD. Computed tomography (CT) imaging appearance of this CHD makes recognition easier.

We discuss a case with truncus arteriosus type IV and the CT imaging.

## CASE REPORT

A 26-year-old female presented with long standing headache and difficulty in breathing on exertion, since childhood. She had peripheral cyanosis with finger clubbing and a palpable systolic murmur. She had an elevated hemoglobin of 22.1 g/dL. Her chest X-ray showed cardiomegaly and transthoracic echocardiography showed features of ventricular septal defect (VSD) and right ventricular hypertrophy. She was sent for further evaluation with a chest CT.

**Figure 1:**
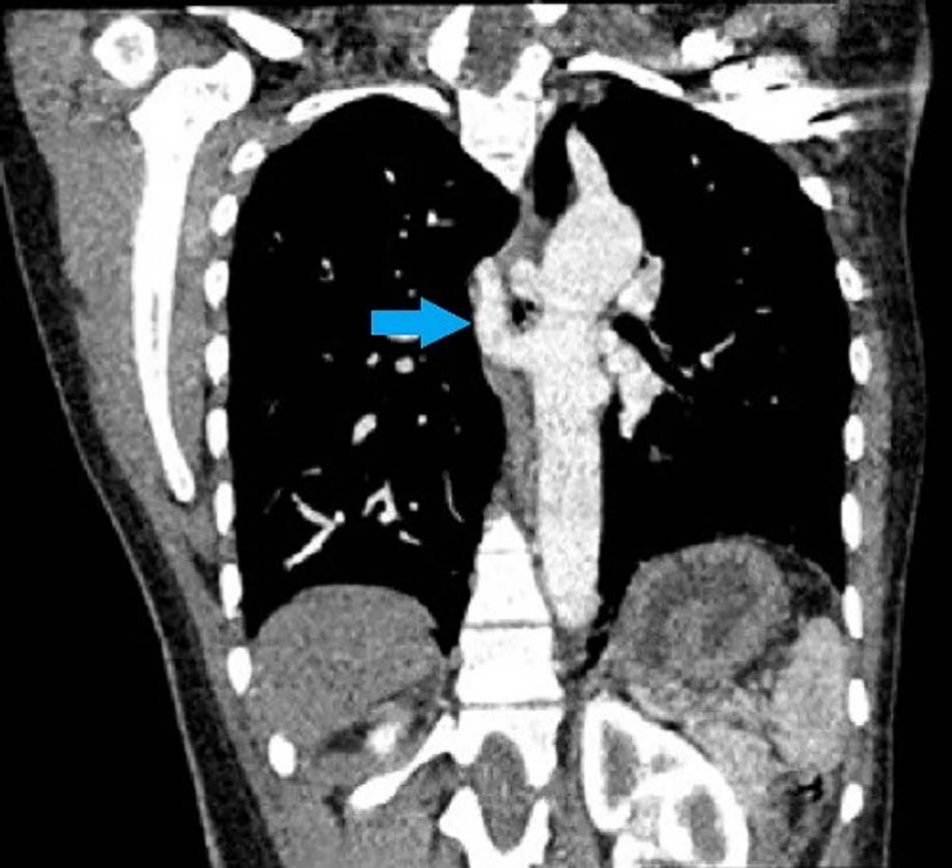
CT chest coronal view shows collaterals (blue arrow) originating separately from the descending aorta.

**Figure 2:**
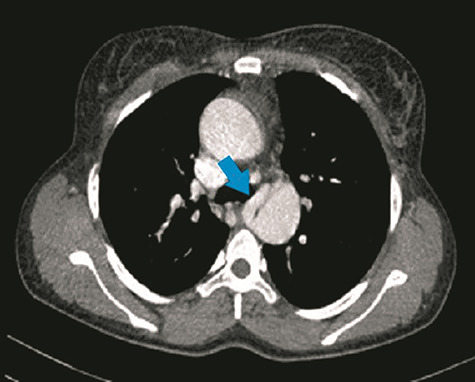
CT chest axial view shows first collateral artery (blue arrow) arising from the right margin of the descending aorta at the level of the carina.

**Figure 3:**
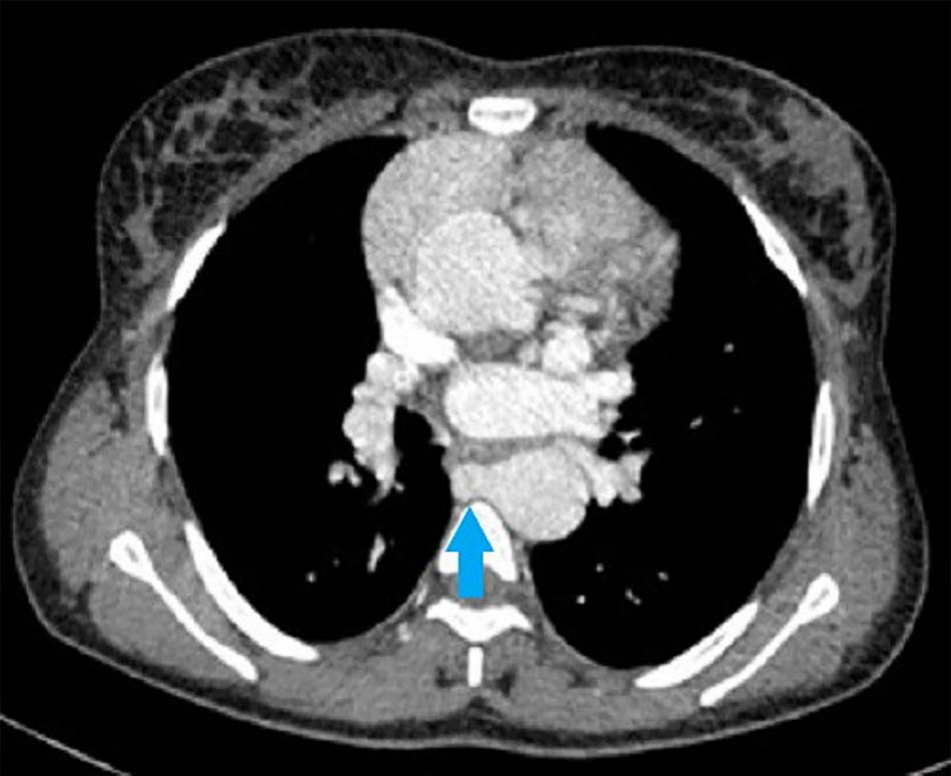
CT chest axial view shows second collateral artery (blue arrow) arising from the right margin of the descending aorta at the level of the left atrium.

**Figure 4:**
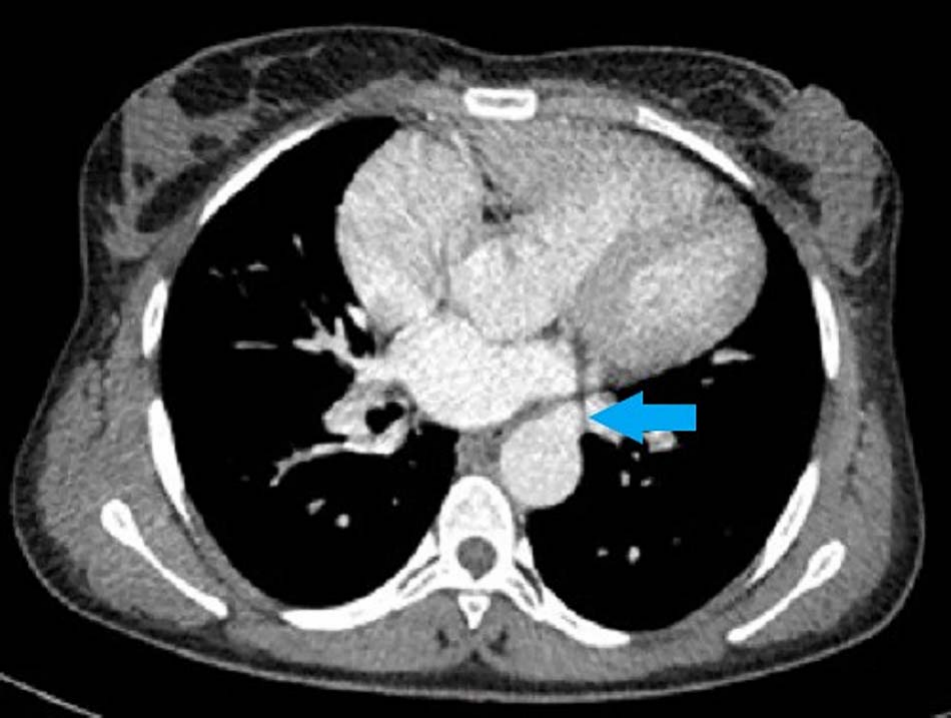
CT chest axial view shows third collateral artery (blue arrow) arising from the left margin of the descending aorta at the level of the left atrium.

Her CT showed cardiomegaly with increased pulmonary vasculature, an overriding aorta and a VSD. The right and left pulmonary arteries originated separately from the descending aorta as seen in Figures 1–4. Reflux of contrast from the right atrium into the hepatic veins and inferior vena cava was suggestive of right heart strain. The parenchyma of both lungs had no significant abnormality. Conclusion of the chest CT showed truncus arteriosus type IV (Collett and Edward classification) with neither pulmonary arterial branch arising from the common trunk (pseudo truncus), also known as pulmonary atresia with a VSD.

She was initially kept on oral furosemide and losartan, with the advice to undergo regular phlebotomy due to the elevated hemoglobin. She was later added on sildenafil and advised to continue with her medication and follow-up.

## DISCUSSION

Africa has fewer number of CHD than other regions. The prevalence of CHD in Africa was around 2 per 1000 births compared with Europe and Asia, which were around 8 and 9 per 1000 births respectively. And the prevalence of PTA was around 7.8 per 100 000 births [2]. In Tanzania, the prevalence of PTA among children with CHD was 3.8% [3], but the type of truncus arteriosus was not known.

There are two methods of classifying PTA as shown in Tables 1 and 2.

**Table 1:** Collett and Edwards classification [4, 5]

Type I	Both aorta and main pulmonary artery arise from a common trunk (48–68% of cases)
Type II	Pulmonary arteries arise separately from the posterior aspect of trunk, close to each other just above the truncal valve (29–48% of cases)
Type III	Pulmonary arteries arise independently from either side of the trunk (6–10% of cases)
Type IV	Neither pulmonary arterial branch arising from the common trunk (pseudo truncus), considered a form of pulmonary atresia with a VSD

**Table 2:** Van Praagh modified classification [6]

Type A1	Both aorta and main pulmonary artery arise from a common trunk (identical to the Type I of Collett and Edwards)
Type A2	Separate origins of the branch pulmonary arteries from the left and right lateral aspects of the common trunk
Type A3	Origin of one branch pulmonary artery from the common trunk, with other lung supplied by collaterals or a pulmonary artery arising from the aortic arch
Type A4	Coexistence of an interrupted aortic arch

In this case, the main pulmonary artery is atretic, and the lung parenchyma is supplied by collaterals from the descending thoracic aorta. The major aortopulmonary collateral arteries (MAPCAs) arise from the junction of the ascending aorta and aortic arch. There is mixing of blood between the ventricles through the overriding aortic valve creating the pulmonary and systemic circulations.

Presence of normal pulmonary arteries causes bilateral pulmonary hypertension unless there is stenosis of either or both pulmonary arteries. Severe stenosis may protect the affected lung but causes segmental pulmonary hypertension on the contralateral lung [7]. Altered perfusion causes ventilation/perfusion mismatch leading to reduced peripheral oxygen delivery, ineffective ventilation and increased workload on the lungs and heart causing right heart failure.

During fetal development, there is an arrest in separation of the anterior pulmonary artery and aorta resulting in a single artery. This may be due to the neural crest cells that contribute to parathyroid and thymus development hence the association between truncus arteriosus and 22q11 deletion, known as DiGeorge syndrome in around 35% of cases [8]. Other factors include teratogenic effects of viral infections, maternal diabetes mellitus in pregnancy, medications and smoking [9].

Truncus arteriosus is characterized by a single vessel from the heart that separates into the ascending aorta and pulmonary arteries in the absence of a pulmonary trunk, whereas in this case, the septation never occurred. It has been suggested that there is no atretic pulmonary valve, and no sub-pulmonary myocardium that represents pulmonary atresia-VSD rather than PTA [8]. Because septation of the great arteries is driven by different neural crest cells, it is possible that septation is partially present because of the reduction in the sub-pulmonary myocardium.

Surgical intervention includes complete repair by closing the VSD and achieving a right ventricle to pulmonary artery connection in children [5]. In cases that have developed pulmonary hypertension, they may undergo a palliative shunt procedure or dilation with or without stenting of the MAPCAs. Medical therapy consisting of endothelin-receptor antagonist bosentan and phosphodiesterase-type 5 inhibitor sildenafil may show some improvement but their use still remains unclear [7].

The oldest reported unrepaired case that survived into adulthood was in the fifth decade [10]. This case is one of the few reported cases that survived into adulthood. The reason could be because of sufficient blood flow to the lungs via collaterals and bilateral bronchial arteries, together with lack of associated congenital malformation [10].

Our case shows a truncus arteriosus type IV, which is a pseudo truncus, classified as pulmonary atresia with VSD, confirmed by CT imaging. With a significant mortality rate within the first year of life of up to 80% [10], our case is one of the few that made it into her 20s.
